# Pars-plana-Vitrektomie – von Saug-Schneide-Systemen bis hin zur Ultraschalltechnik: Vitesse – eine neuartige Form der Vitrektomie mittels einer Ultraschalltechnik

**DOI:** 10.1007/s00347-021-01377-6

**Published:** 2021-04-20

**Authors:** Svenja Deuchler, Timo Knoch, Asael Papour, Thomas Kohnen, Frank Koch

**Affiliations:** 1 Augenklinik, Goethe-Universität Frankfurt, Theodor-Stern-Kai 7, 60590 Frankfurt am Main, Deutschland; 2 Bausch + Lomb GmbH, Brunsbütteler Damm 165/173, 13581 Berlin, Deutschland

**Keywords:** Vitrektomie, Liquifizierung, Glaskörper, Ultraschall, Operation, Vitrectomy, Liquification, Vitreous body, Ultrasound, Surgery

## Abstract

Um 1970 herum wurde erstmals ein Schneidegerät, in welches ein Infusions- und ein Aspirationskanal integriert wurden – der „vitreous infusion suction cutter“ (V.I.S.C) –, standardmäßig für die Entfernung des Glaskörpers angewandt. Schneidesysteme haben sich von da an immer weiter entwickelt. Schallenergie blieb lange den Vorderabschnittschirurgien vorbehalten. Ab 2020 kann in Form der Vitesse™ Schall auch für den Hinterabschnitt genutzt werden. Bei diesem Verfahren der Liquifizierungstechnik wird Ultraschallenergie auf ein einzelnes, großes inneres Lumen übertragen, das den Port der Nadel mechanisch in Schwingung versetzt. Diese Hochgeschwindigkeitsvibrationen scheren den Glaskörper an den Portkanten ab, bevor er in die Nadel eintritt. Dieser Mechanismus minimiert die Traktion des Glaskörpers und ermöglicht eine schnellere Aspiration bei niedrigerem Vakuum im Vergleich zu herkömmlichen Schneidesystemen. Diese Vorteile können zu einer sichereren und effizienteren vitreoretinalen Chirurgie führen.

**Video online:**

Die Online-Version dieses Beitrags (10.1007/s00347-021-01377-6) enthält Videos.

## Abstract (en)

In the 1970s a cutting device into which an infusion and an aspiration channels were integrated, the so-called vitreous infusion suction cutter (V.I.S.C), was used for the first time for the removal of the vitreous body. These cutting systems have continued to constantly evolve since then; however, sonic energy remained reserved for anterior segment surgery for a long time. In 2020, this form of energy could also be used for the posterior segment in form of Vitesse™. In this liquification technique, ultrasonic energy is transferred to a single, large inner lumen design that mechanically vibrates the needle’s port. These high-speed vibrations shear the vitreous body at the port edges before it enters the needle. This mechanism reduces traction to a minimal level and enables faster aspiration at lower vacuum levels compared to conventional cutters. These advantages can promote a safer and more efficient vitreoretinal surgery.

## Entwicklungsschritte der Glaskörperschneidetechnologie [3, 4]

Robert Machemer, sein Ingenieur Dyson Hickinbotham und Jean Marie Parel [9] entwickelten Anfang der 70er-Jahre die „geschlossene“ Pars-plana-Technologie (PPV), eine Antwort auf die vielfältigen Risiken der bis dahin durchgeführten Open-sky-Strategie [2], bei der – allen voran – Hypotonie, Aderhautschwellung und Blutungen in die Augenwand und das Augeninnere zu desaströsen Komplikationen zählten. Damals tat David Kasner den entscheidenden Ausspruch, der wahrscheinlich Antriebe für die Entwicklung der Vitrektomie im „geschlossenen“ Auge war: „Der Glaskörper ist der größte Feind des Kataraktchirurgen“ [8]. Auch erübrigten sich damit Manipulationen an der Hornhaut, wie die Verwendung einer temporären Keratoprothese sowie das Procedere der Keratoplastik. Das Verfahren der Vitrektomie war somit deutlich weniger invasiv für den Bulbus. Unter Machemers Führung wurden die Entwicklungen auch klinisch angewandt, und zwar zunächst unter Einsatz eines Schneidegeräts, in welches ein Infusions- und ein Aspirationskanal integriert wurden, der sog. „vitreous infusion suction cutter“ (V.I.S.C). Dass zuvor bzw. parallel hierzu offenbar ähnliche Entwicklungen in Japan stattgefunden hatten und dass Anton Banko ein Gerät mit diesem Prinzip hatte patentieren lassen, ist für uns heute von untergeordneter Bedeutung, denn erst durch die kommerzielle Verbreitung des „V.I.S.C.“ kam es zur angewandten Revolution der PPV. Hält man sich vor Augen, dass wir heute in 2020 Schalltechnologie für die Glaskörperentfernung entwickeln, ist es umso bemerkenswerter, dass Banko in den 70ern neben der Aspiration/Infusion für einen Glaskörpercutter damals die Fluidics für die initiale Phakomaschine von Charles Kelman zu verantworten hatte. Im Rahmen mechanischer Linsenoperationssysteme sowie zu Beginn des Einsatzes von Phakotechnologie wurde ihm nur allzu häufig die Problematik mit dem unerwünschten Kontakt zu Glaskörper im Rahmen von Linsenchirurgie vorgeführt. Dennoch blieb Schallenergie noch lange Zeit den Vorderabschnittschirurgen vorbehalten. Zur Komplettierung des V.I.S.C.-Schneidegeräts entwickelte Jean Marie Parel fiberoptische Endoilluminatoren und den (infusions- und aspirationsunabhängigen) Membran-Peeling-Cutter (MPC), ein vertikal arbeitendes Schneidegerät, welches elektronisch angetrieben und später auch in Varianten manuell zum Einsatz kam. Dadurch, dass sich mit dem MPC „nur“ hervorragend Gewebeschnitte durchführen lassen konnten und Infusion und Aspiration über weitere Zugänge und zusätzliche Gerätschaften stattfanden, mussten mehrere Augenöffnungen angelegt werden. Aber statt einer Öffnung für ein Gerät mit Außendurchmesser von bis zu 2,3 mm konnten nun Öffnungen von weniger als 1 mm angelegt werden. Schnell wurde klar, dass mehrere kleine Öffnungen mit einer deutlich besseren Tonisierung des Augapfels einhergehen. Da der V.I.S.C. als „rotierender Cutter“ durch das Rotationsprinzip eine Aufspulung von Glaskörperfasern vor deren Schnitt – und damit eine unerwünschte Traktion des Glaskörpers an der Netzhaut – erzeugen konnte, schuf Nicholas Douvas in Zusammenarbeit mit Machemer den sog. „RotoExtractor“ – ähnlich multifunktionell wie der V.I.S.C. –, aber mit einem Oszillations-Mode ausgestattet, der die Glaskörperaufwicklungs- und -spannungsgefahr reduzieren sollte. Conor O’Malley und Ralph Heinz stellten der 3‑Port-PPV eine leichten, wiederverwendbaren, pneumatischen, sich „axial“ bewegenden Cutter zu Verfügung, welcher auf der sog. Ocutome 800 Konsole (Berkley Bioengineering, 1972) betrieben wurde. Zeitgleich kam es dann zur Einführung elektrischer, axial arbeitender Guillotinen – Schneidegeräte – durch Gholam Peyman und R. Klöti, deren Arbeitsweise und Größenmaße lange Zeit den Goldstandard der 20-Gauge-3-Port-Pars-plana-Vitrektomie darstellten.

Die Errungenschaften im Bereich der Materialkunde sind Ausgangspunkt für weitere Schritte auf dem Weg zur Minimalisierung des chirurgischen Traumas, erzielt durch eine Mehr-Port-Pars-plana-Vitrektomie:

Durch Verbesserung der Stabilität – v. a. des Instrumentenschafts – lässt sich der Standard der Glaskörperchirurgie von 0,9 mm auf 0,6 und 0,5 mm (und kleiner) reduzieren – wobei die Maße nach Einführen der transkonjunktivalen „nahtlosen Glaskörperchirurgie“ [7] dem Außendurchmesser von Kanülen entsprechen. Somit müssen die Instrumente noch etwas kleiner gestaltet sein, um durch den jeweiligen Kanülenstandard (gemäß der jeweiligen Gauge-Angabe, z. B. 23 Gauge) in das Augeninnere eingeführt werden zu können.

Die anfänglich hierdurch erschwerten Schneide- und Transportabläufe wurden durch Veränderungen des Klingendesigns und der Portgeometrie in den Schneidegeräten kompensiert. Durch Wahl hoher Schneidefrequenzen wird bei Einsatz von Vitrektomiegeräten mit Venturi-Pumpe die Aggressivität des Schnitts in direkter Nähe der Netzhaut herabgesetzt und besser kontrollierbar, der Einsatz neuer Klingendesigns steigert die Effizienz der Glaskörperentfernung in einer gleichzeitig sicheren Umgebung. Vordergründiges Ziel hierbei ist, bei steigender Geschwindigkeit der Schneide keinen Verlust der Flussraten in Kauf zu nehmen, sodass bei kleineren Schnitten die gleichen Flussraten und damit Zu- und Abflussgeschwindigkeiten gewährleistet sind.

Der MID Labs Bi-Blade™ Vitrektomie-Cutter (Bausch&Lomb, St. Louis, MO, USA) hat eine zusätzliche Schneide, die es ermöglicht, in einer einzigen Bewegung 2‑mal zu schneiden (7500 cpm × 2). Die Schneideöffnung ist weitgehend kontinuierlich offen und die Aspirationsrate somit unabhängig von der Schneidrate: Das schafft Geschwindigkeit, Sicherheit und Stabilität für das gesamte Verfahren.

## Ultraschalltechnik mittels Vitesse [5]

### Hintergrund

**Figure 1:**
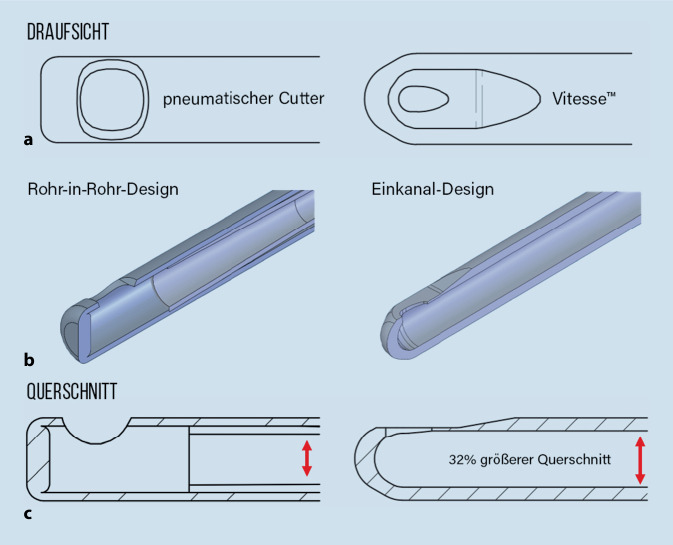

Vitesse™ (Bausch&Lomb, St. Louis, MO, USA) arbeitet in Richtung Optimierung von Geschwindigkeit, Sicherheit und Stabilität. Vitesse™ stellt eine Abkehr von der traditionellen Vitrektomietechnologie dar und bietet neue Möglichkeiten, die Operationen zu rationalisieren und optional die Ergebnisse für den Patienten zu verbessern. Das Vitesse™-Handstück (Abb. 1) basiert auf grundlegenden Prinzipien, gepaart mit einem innovativen Design, das es ermöglicht, eine einlumige Nadel mit offenem Seitenanschluss an einen Ultraschallwandler anzuschließen. Die Energie des Schallkopfes liefert eine fokussierte Gewebeschneidefähigkeit am Port nahe der Nadelspitze. Der Wirkungsmechanismus kann als „mechanische Ultraschallscherung“ beschrieben werden, da die Portwände in Längsrichtung mit Ultraschallfrequenzen schwingen. Wenn im Auge operiert wird, greift der Nadelport in den Glaskörper ein und schert ihn mit einer Schnittrate, die Millionen Schnitten pro Minute entspricht. Während der Glaskörper durch den Port läuft, hat der aspirierte Glaskörper nun seine Eigenschaften dramatisch verändert. Die langen Kollagenfibrillen, die für die mechanischen Eigenschaften des Glaskörpers verantwortlich sind, sind in mikroskopisch kleine Fragmente zerfallen, wodurch die Glaskörperfestigkeit und die scheinbare Viskosität deutlich abnehmen. Die Vorteile dieses Mechanismus sind vielfältig. Er ermöglicht eine kontinuierliche, ununterbrochene und effiziente Absaugung aufgrund des 100 % offenen Port-Duty-Cycle. Dies wiederum ermöglicht eine reibungslose Fluidics-Wirkung, da es keine mechanische Unterbrechung durch einen inneren Nadelschneider wie bei pneumatischen Schneidegeräten gibt. Ein weiterer wichtiger Aspekt der Technologie ist der reduzierte Zug am Glaskörper: Er wird vor der Mündung des Ports durchtrennt, also bevor er in das innere Nadellumen eintritt, das im Gegensatz zu pneumatischen Cuttern zuerst aspiriert und dann den Glaskörper durchtrennt, nachdem Zug entstanden ist.

## Derzeitiger Stand der Vitesse

Der Ursprung der Entwicklung der Vitesse™ Technologie geht zurück auf das Jahr 2011, in welchem Dr. Blinder [1] in Zusammenarbeit mit Bausch & Lomb die Perkins-Multiportnadel näher evaluierte. Hier zeigte sich, dass die Ultraschalltechnologie auch für die Entfernung von Glaskörpergewebe attraktiv sein könnte und die präklinische Forschung begann, um u. a. Fragen wie der idealen Lage der Aspirationsöffnung, deren Form sowie den vielfältigen Sicherheitsaspekten nachzugehen. Die erste klinische Studie wurde dann erfolgreich in Indien im Jahr 2017 durchgeführt. Kurz darauf erfolgte die FDA-Zulassung und die erste klinische Anwendung in den USA im September 2018. Im gleichen Jahr erfolgte die CE-Zertifizierung für den europäischen Anwendungsbereich.

## Unterschiede in den Parametereinstellungen

Bei dem derzeitigen Goldstandard in der Vitrektomie wird der Glaskörper mittels eines pneumatisch betriebenen Vitrektoms zuerst in ein Saug- und Schneidrohr aspiriert und danach zerkleinert (Guillotinen-Prinzip). Bauartbedingt soll mit Vakuumwerten von ca. 200 mm Hg bis 600 mm Hg gearbeitet werden, damit dies effektiv durchgeführt werden kann, und die Zerkleinerung findet im Saug-Schneid-Rohr statt. Dies hat zur Folge, dass gerade bei der Entfernung des kompletten Glaskörpers an der Glaskörperbasis Traktion entstehen kann bis hin zur Erzeugung von iatrogenen Foramina.

Die Vitesse™ wendet ein neuartiges Verfahren der Liquifizierungstechnik an. Hier wird durch Schwingungen einer Hohlnadel (0–60 µm bei 28,5 kHz) und Erzeugung von Scherkräften in deren Umfeld der Glaskörper bereits vor der Aspirationsöffnung liquifiziert und kann somit bei wesentlich niedrigeren Vakuumwerten (0–150 mm Hg) abgesaugt werden. Durch diese reduzierten Vakuumwerte verringert sich die Traktion, und da die Liquifizierung bereits vor der Portöffnung stattfindet, kann in der Peripherie an der Glaskörperbasis der Glaskörper sicherer und vollständiger entfernt werden.

## Klinische Anwendungen der Liquifizierungstechnik durch Vitesse

Die Liquifizierungstechnik mittels Vitesse™ kann bei allen Vitrektomien als Alternativverfahren zur herkömmlichen Saug-Schneide-Technik angewendet werden.

### Vorteile

**Figure 2:**
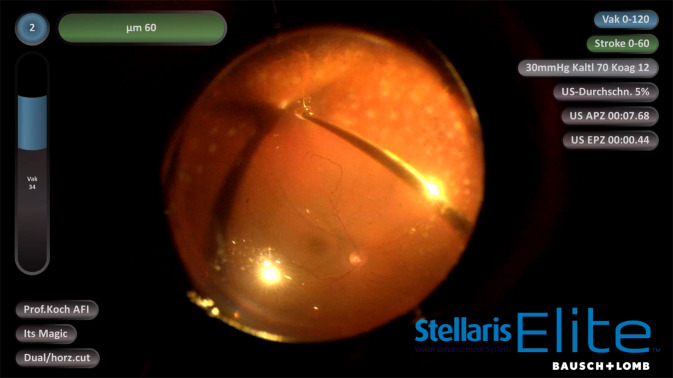

**Figure 3:**
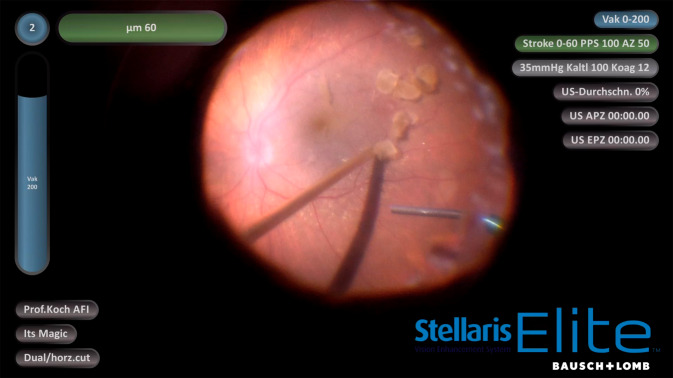

Wie bereits oben aufgeführt, ist ein Vorteil der Ultraschalltechnik die Verringerung der Traktionserzeugung: Anknüpfend an die Weiterentwicklung bestehender pneumatischer Guillotinen-Cutter z. B. in Form des Doppelklingen-Cutters (Bi-Blade), bei dem, bezogen auf die Öffnungszeit des Cutters, pro Ansaugzeit mehr Gewebe geschnitten wird (Anstieg der effektiven Schneidrate), bedeutet die Liquifizierung durch die Vitesse™, dass jegliches Ansaugen von Gewebe in ein Schneidrohr ohne vorangegangene Verflüssigung völlig entfällt („external mechanical shearing“). Daraus ergeben sich unter anderem folgende Gesichtspunkte:

- Die Zunahme an Sicherheit bei der Entfernung des Glaskörpers von der Netzhaut an der Glaskörperbasis, nicht nur unter Flüssigkeit, sondern auch unter Luft (Abb. 2) mit dem Ausbleiben der Gefahr einer Hypotonieentwicklung, welche sonst bis hin zum Kollaps eines Auges führen könnte, sowie von unerwünschten iatrogen erzeugten Netzhautlöchern. Die Vitrektomie nach Flüssigkeit-zu-Luft-Austausch ist v. a. dann angezeigt, wenn flottierende Netzhautstrukturen vor Wiederanlage der Retina die optisch kontrollierte und technisch sichere Gewebemanipulation zu verhindern drohen.
- Einfache Glaskörperabhebung bis in die Peripherie: Mit der Vitesse-Technologie wird die Glaskörperentfernung vom hinteren Pol bis in die äußerste Peripherie gefahrenarm möglich. Dies umfasst auch die präzise Manipulation bei Bestehen einer vitreoretinalen Schisis in der Makula. Weiterhin ist die Beseitigung von Glaskörper vor einer bullös abgehobenen Netzhaut ohne Erzeugung von ungewollten Drainagelöchern zuverlässig möglich.
- Die gezielte Behandlung von Retinotomielöchern: Die Lochkantenauffrischung (Beseitigung von nekrotischem Geweberand) kann ohne unzureichend kontrollierte Vergrößerung eines bestehenden Defektes durchgeführt werden. Dabei kann der Umfang der Gewebeentfernung gut eingeschätzt werden, weil er pro Aktion am Geweberand durch die Öffnung der Vitesse-Sonde definiert ist, mit anderen Worten: Das Gewebe wird mit ca. 250 Micron Präzision abgetragen.
- Glasköperblutungen, Fibrin und Pigmentzellen können kontrolliert entfernt werden. Dies ist dann von größter Bedeutung, wenn z. B. bei einer PVR-Amotio retinae diese Zellen ohne Erzeugung eines Netzhautdefektes nah auf der Oberfläche entfernt werden müssen. Mit herkömmlichen Guillotine-Cuttern nimmt der Chirurg meist Abstand von diesem Vorhaben, weil er befürchtet, unkontrolliert Netzhautlöcher zu erzeugen, und dann das Zusammentreffen von Loch und verbliebenen Zellen den Vorgang einer proliferativen Vitreoretinopathie (PVR) anheizt.
- Ein weiteres neues Einsatzgebiet für die Liquifizierungstechnik ist die Entfernung von epiretinalen Membranen, welche bisher mechanisch durch den Operateur vornehmlich unter Verwendung mikrochirurgischer Pinzetten entfernt werden mussten. Das Handstück für die Liquifizierungstechnik kann den Operateur sowohl bei der jetzigen Technik unterstützen (Anheben von Membrankanten zum einfacheren und sicheren Greifen der Membrane) als auch neue Möglichkeiten (durch Liquifikation vor der Portöffnung) bei dieser Pathogenese bieten.
- Die Bergung eines „versenkten“ Linsenkerns nach komplizierter Kataraktoperation ist äußerst effizient möglich (Abb. 3). Hier ist es am effektivsten, die Linsenfragmente zunächst mit reinem Vakuum anzusaugen und dann nach der Wahl des sog. „pulse modes“ (duty cycly/DC 30–50 %) und mittels eines bimanuellen Procederes (Vitesse und Pinzette) diese anzusaugen. Der bimanuelle Ansatz hilft v. a., Kernanteile vor der Vitesse-Mündung optimal zu positionieren, sodass diese zügig verflüssigt werden können.

### Nachteile

- Relativ „nachteilig“ zeigt sich, dass beim derzeitigen Aufbau des Handstücks die Liquifizierungstechnik sicherer, aber auch etwas zeitaufwendiger ist, und zwar bei der Core-Vitrektomie. Hierfür wird kurzfristig eine Lösung angeboten (Sommer 2021).
- Die Trokare sind systembedingt aus Kunststoff (Polyamid vs. Titan). Hier zeigt sich, dass sich die Plastikkanülen etwas leichter aus der Eingangsstelle abstoßen, mit anderen Worten, sie sitzen etwas weniger fest in der Augenwand. Hier wurde von den Autoren angeregt, die Oberfläche der für das Vitesse-Handstück notwendigen Kanülen zu modifizieren.
- Bestimmte Konstellationen von Zellen und Gewebe bei blutbetauten Glaskörpersträngen sind in Wasser für Vitesse zum Teil ein schwieriges Unterfangen. Hier kann es aufgrund des eher „sanfteren Verfahrens“ zu einer Verstopfung des Ports bei dichtem Glaskörperblut bzw. einer zähen Membran kommen, insbesondere bei deren Aufeinandertreffen. Es hat sich im kontrollierten, klinischen Einsatz gezeigt, dass ein Verharren auf der Stelle („sich in Geduld üben“) unter gleichzeitiger Reduktion des Vakuums helfen kann. Alternativ können die Membranen auch bimanuell bzw. nach Flüssigkeit-zu-Luft-Austausch entfernt werden: Dabei funktioniert die Membranentfernung am besten ohne Indentation des Bulbus.

## Konkrete Tipps und Tricks aus der klinischen Erfahrung für den Einsatz von Vitesse

- Bei der Core-Vitrektomie sollte man zur Verbesserung der Effizienz den maximalen Stroke (60 Micron) und ein Vakuum von bis zu 200 mm Hg (bevorzugt bei dual-linearer Steuerung) einsetzen.
- Subretinales Blut allein mit Vakuum aspirieren (kein Stroke erforderlich).
- Bei Erzeugung von unerwünschten „vitreous perls“ ist es sinnvoll, das Vakuum zu reduzieren und an einer Stelle zu verharren, um der Vitesse etwas mehr Zeit zu geben (cave: Hier muss man lernen, sich „in Geduld zu üben“, langsame Bewegungen durchzuführen).
- Arbeiten auf der Fovea sind bei vollem Vakuum und 50 % Stroke sicher. Das gilt für ERM-Peeling auch bei unmittelbarer Nähe z. B. zu fovealen Zysten.
- Um einen Rückstoß von Luft(blasen) oder von gepeelten ILM-Membranen zu vermeiden, sind diese aus dem Glaskörper am einfachsten zu entfernen, wenn diese erst mit Vakuum ohne Stroke angesaugt werden und dann konsekutiv ein geringer Stroke von ca. 20 Micron dazugeschaltet wird.

## Kritische Abwägung bestehender Ansätze und Blick in die Zukunft

In Zukunft könnte die Option genutzt werden, aufgrund des Einkanalsystems (Innenvolumen größer als bei Guillotine-Cutter) die Instrumentensonde auch in gebogenem Zustand zum Einsatz kommen zu lassen. Weil Vitesse™ jedes Gewebe in jedem Medium (z. B. Linsenanteile, egal wie hart, etc.) beseitigen kann, wird sie so zunehmend unabhängig von weiteren Instrumenten: Das bedeutet, dass die weiteren Zugänge bei einer 4‑Kanal PPV für andere Instrumente mit neuen Zusatzfunktionen freigestellt werden bzw. statt einer 4‑ evtl. eine 3‑ oder sogar 2‑Kanal-Technologie gewählt werden kann. Ob die geringeren Vibrationsauswirkungen und die „schweigsamere“ Geräuschkulisse der Vitesse vs. z. B. des Bi-Blade-Cutters tatsächlich vorteilhaft sein werden, könnte von ChirurgIn zu ChirurgIn unterschiedlich bewertet werden. Die oben aufgeführten „Tipps und Tricks“ sollten von allen Benutzern wahrgenommen und berücksichtigt werden – erst dann werden wir lernen, welche Neuartigkeiten die Vitesse-Ultraschalltechnik tatsächlich hervorbringt und welche davon klinisch relevant sein werden. Unsere eigenen langjährigen Studien haben in 2018 erlaubt, basierend auf herkömmlichen „Schnitt-Liquifizierungstechnologien“, bestimmte SOPs für die Netzhaut- und Glaskörperchirurgie zu entwickeln, welche vor allem bei aufwändigeren Fällen wie Proliferativer (Diabetischer) VitreoRetinopathie (PVR, PDVR) mit zum Gelingen des Operationsergebnis beitragen [6]. Wir gehen davon aus, dass mithilfe bestehender Evaluationsverfahren bei Analyse der neuartigen Vitesse-Schall-Technologie neue „Key-Factors“ für den Erfolg der Glaskörperchirurgie erarbeitet werden können.

Ob mittel- bis langfristig die Vitesse™-Technologie weiter subsumiert werden sollte unter „Vitrektomie“ (lateinisch „vitreus“ „gläsern“, griechisch „ek“ „heraus“ und „tomein“ „schneiden“) oder die wörtliche Übersetzung des „Glaskörper herausschneiden“ durch eine neue Begrifflichkeit abzulösen ist, wird abzuwägen sein. „Glaskörper-Verflüssigung mittels Vitesse™“ würde der Tatsache gerecht, dass es zu keinem Zeitpunkt zu Zugauswirkungen auf nicht aufbereitete Glaskörperstränge kommt.

## Fazit für die Praxis

Der Einsatz der Ultraschalltechnologie in der Glaskörperchirurgie vereinfacht viele Eingriffsschritte. Wo Vereinfachungen vorliegen und wo alternative Maßnahmen den zusätzlichen Einsatz weiterer Systeme sinnvoll machen, scheint heute schon absehbar zu sein – es sind allerdings weitere Erkenntnisse im zunehmenden Umgang mit Vitesse™ und deren Weiterentwicklung zu erwarten. Sollten sich die bisherigen Erkenntnisse und Entwicklungen in dieser Form fortsetzten, dann kann die Vitesse-Ultraschalltechnologie zum Goldstandard in der Glaskörperchirurgie avancieren, v. a. dann, wenn das Sicherheitsprofil unter Erhalt oder Verbesserung der Effizienz zunimmt.

## Supplementary Information




