# Erratum zu: Pars-plana-Vitrektomie – von Saug-Schneide-Systemen bis hin zur Ultraschalltechnik: Vitesse – eine neuartige Form der Vitrektomie mittels einer Ultraschalltechnik

**DOI:** 10.1007/s00347-021-01431-3

**Published:** 2021-06-14

**Authors:** Svenja Deuchler, Timo Knoch, Asael Papour, Thomas Kohnen, Frank Koch

**Affiliations:** 1 Augenklinik, Goethe-Universität Frankfurt, Theodor-Stern-Kai 7, 60590 Frankfurt am Main, Deutschland; 2 Bausch+ Lomb GmbH, Berlin, Deutschland

**Erratum zu:**

**Ophthalmologe 2021**

10.1007/s00347-021-01377-6

In dem ursprünglichen Artikel war der Interessenkonflikt des Autors Thomas Kohnen unvollständig angegeben. Die Online-Version sowie das PDF des Beitrags wurden nachträglich korrigiert. Bitte beachten Sie diese Änderung.

Der vollständige und korrigierte Artikel steht Ihnen auf www.springermedizin.de zur Verfügung. Bitte geben Sie dort den Beitragstitel in die Suche ein.

Die Redaktion

